# A review of bronchiolitis obliterans syndrome and therapeutic strategies

**DOI:** 10.1186/1749-8090-6-92

**Published:** 2011-07-18

**Authors:** Don Hayes

**Affiliations:** 1 The Ohio State University Columbus, OH, USA

## Abstract

Lung transplantation is an important treatment option for patients with advanced lung disease. Survival rates for lung transplant recipients have improved; however, the major obstacle limiting better survival is bronchiolitis obliterans syndrome (BOS). In the last decade, survival after lung retransplantation has improved for transplant recipients with BOS. This manuscript reviews BOS along with the current therapeutic strategies, including recent outcomes for lung retransplantation.

## Introduction

**Figure 1:**
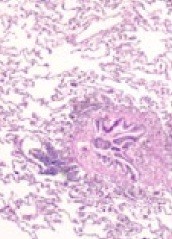
**Representative histopathology of obliterative bronchiolitis with inflammation and fibrosis of the airway with sparing of the surrounding alveoli (Hematoxylin and Eosin stain)**.

Lung transplantation is a treatment option for patients with advanced lung disease or irreversible pulmonary failure. Despite advancements in surgical techniques, lung preservation, immunosuppression, and management of ischemia/reperfusion injury and infections, acute and chronic allograft rejection continues to be a major problem. The incidence and severity of acute rejection in lung transplantation exceeds all other solid organ transplants [1,2]. Chronic rejection, more commonly called bronchiolitis obliterans syndrome (BOS), is the leading cause of death beyond the first year post lung transplantation [3,4]. The key clinical feature of BOS is the development of airway obstruction with a reduction of forced expiratory volume in 1 second (FEV_1_) that does not respond to bronchodilators (Table 1) [5,6]. The hallmark histological findings of chronic rejection is obliterative bronchiolitis (OB), which is an inflammatory process affecting small noncartilagenous airways [7,8]. Figure 1 is representative of the typical findings of OB histopathologically. The development of BOS is rare within the first year after lung transplant, but the cumulative incidence ranges from 43 to 80% within the first five years of transplantation [4,9-11].

**Table 1:** Bronchiolitis obliterans syndrome (BOS) classification

BOS Stage	Classification
0	FEV_1 _> 90% of baseline & FEF_25-75% _> 75% of baseline

0-p*	FEV_1 _81-90% of baseline &/or FEF_25-75% _≤ 75% of baseline

1	FEV_1 _66-80% of baseline

2	FEV_1 _51-65% of baseline

3	FEV_1 _≤ 50% of baseline

*0-p = potential BOS, Modified from Reference #6

## Diagnosis

The diagnosis of BOS is typically made by clinical, physiological, and radiographic parameters. Due to the sporadic or patchy involvement of OB, pathologic diagnosis can be missed by transbronchial biopsies (TBB) [5], which are often performed to exclude other diagnoses including acute rejection or infection. Histologically, early lesions of BOS demonstrate submucosal lymphocytic inflammation and disruption of the epithelium of small airways, followed by an ingrowth of fibromyxoid granulation tissue into the airway lumen, resulting in partial or complete obstruction. Subsequently, granulation tissue organizes in a cicatricial pattern with resultant fibrosis and thus obliterates the airway lumen [12]. In some instances, the only residual histologic evidence of BOS is a ring of circumferential elastin around an otherwise undetectable airway, what is termed the "vanishing airways disease" [12].

As a result of histologic variability, the International Society for Heart and Lung Transplant (ISHLT) developed standard nomenclature and made a distinction between documented OB and BOS [13]. An ad hoc working group was established under the auspices of the ISHLT for the purpose of developing a clinically applicable system and published their original recommendations in 1993 [13]. The group concluded that the FEV_1 _was the most reliable and consistent indicator of allograft dysfunction, excluding other identifiable causes with the adoption of the term BOS to describe such dysfunction, recognizing that there may or may not be pathologic evidence of OB present [13]. The group also defined 4 stages of BOS, each with 2 subcategories to indicate whether pathologic evidence of OB had been identified [13].

The clinical course of BOS can vary from insidious onset and gradual decline in pulmonary function over months to years to abrupt onset with severe decline in pulmonary function over a few weeks [14-16]. The clinical diagnosis of BOS requires a sustained pulmonary decline with a reduced FEV_1 _for more than 3 weeks and the exclusion of acute allograft rejection, anastomotic complications or stricture, infection, or other disease affecting pulmonary function. In comparison, acute allograft rejection is defined as perivascular or peribronchial mononuclear inflammation that may be associated with an acute reduction in pulmonary function. Clinical presentation of acute allograft rejection may vary from asymptomatic patients with acute rejection found on surveillance biopsy to non-specific symptoms including cough, dyspnea, sputum production, fever, hypoxia, and adventitious sounds on lung auscultation [8,15]. The current classification of BOS is based on changes in FEV_1 _with the maximum post-transplant FEV_1 _being assigned a 100% predicted value (the mean of the two best postoperative FEV_1 _values with at least 3 weeks between the measurements) and the reduction in the mean forced expiratory flow during the middle half of the forced vital capacity (FEF_25-75%_) used as an early marker for BOS or potential BOS [5]. The current ISHLT classification system for BOS is outlined in Table 1.

Currently, radiographic imaging is not used as a diagnostic tool in transplant recipients when evaluating for BOS; however, high resolution computed tomography (HRCT) imaging with inspiratory and expiratory views may be helpful when considering the diagnosis. Numerous abnormalities may be seen including hyperlucency or air-trapping, bronchiectasis, thickening of septal lines, mosaic pattern of attenuation, or tree-in-bud pattern [17]. Obtaining an expiratory CT scan may help reveal air-trapping that is not evident on inspiratory scans in BOS [17,18]. Furthermore, the extent of air-trapping may correlate with BOS severity [18].

## Pathogenesis and Risk Factors for BOS

The pathogenesis of BOS is complex and involves both alloimmune and non-alloimune mechanisms that occur alone or in combination. Chronic rejection is classified pathologically as either chronic vascular rejection or chronic airway rejection [7]. Chronic vascular rejection, the less common manifestation of rejection, involves the development of atherosclerosis in the pulmonary vasculature [7]. Chronic airway rejection, which is defined as OB histologically, is seen more frequently and results in increased morbidity and mortality [7,19]. Table 2 summarizes the current reported risk factors associated with the development of BOS in lung transplant recipients. The major risk factors associated with BOS are reviewed in the following paragraphs.

**Table 2:** Risks factors for bronchiolitis obliterans syndrome after lung transplantation.

Probable	Potential
Acute rejection	*Aspergillus *colonization of the lower airways

CMV pneumonitis	Aspiration

HLA-mismatching	CMV infection (without pneumonitis)

Lymphocytic bronchitis/bronchiolitis	Donor antigen-specific activity

Noncompliance with medications	Epstein-Barr virus reactivation

Primary graft dysfunction	Etiology of native lung disease

	Gastroesophageal reflux

	Older donor age

	Pneumonia (gram negatives, gram positives, fungi)

	Prolonged allograft ischemia

	Recurrent infection other than CMV

### Acute rejection

Acute rejection is well defined as a primary risk factor in the development of BOS [9,20-25]. Recurrent, late, and severe episodes of acute rejection have all been associated with an increased risk for BOS. Moreover, Hachem et al [26] recently demonstrated that a single episode of minimal acute rejection without recurrence or progression to a higher grade of rejection was a significant predictor of BOS independent of other risk factors.

### Pneumonia/Airway colonization

Pneumonia and/or airway colonization with gram positive and gram negative pathogens as well as fungi are independent determinants of chronic allograft dysfunction [27]. In an interesting study, serology to *Chlamydia pneumoniae *in donors and recipients was associated with the development of BOS in lung transplant recipients. In fact, BOS occurred more frequently and earlier in *C. pneumoniae *seropositive donors, and the reverse was true in seronegative recipients [28]. In another study, colonization of the lower airways with *Aspergillus *was also determined to be a potential causative role for the development of BOS post-lung transplantation [29]. Exudative bronchiolitis, as determined by HRCT imaging, was associated with an increased risk of BOS in lung transplant recipients [30].

### Type of transplant

The type of transplant, whether single or bilateral, may be a risk factor for the development of BOS. In a retrospective review of 221 lung transplant recipients with chronic obstructive pulmonary disease (COPD), bilateral transplant recipients were more likely to be free of BOS than single recipients three years (57.4% vs 50.7%) and five years (44.5% vs 17.9%) after transplantation (P = 0.024) [31].

### Viral infection

Lower respiratory tract infections due to community acquired respiratory viruses have been reported to increase the risk for BOS, including rhinovirus, coronavirus, respiratory syncytial virus, influenza A, parainfluenza, human metapneumovirus, and human herpes virus-6 [32-35]. Therefore, treatment of these viral infections theoretically may reduce the incidence of BOS, but data are limited [36]. Cytomegalovirus (CMV) infection has also been well described as a potential risk factor in the development of BOS; [19,37,38] however, one study demonstrated that histopathologically confirmed CMV pneumonia treated with ganciclovir was not a risk factor for BOS or patient survival nor was any particular CMV serologic donor/recipient group [39]. The treatment of CMV and the subsequent prevention of BOS remains unclear. In a more recent study, Epstein-Barr virus (EBV) reactivation detection by repeated EBV DNA analysis of blood in lung transplant recipients was associated with the development of BOS [40].

### Primary graft dysfunction

Ischemia-reperfusion injury after lung transplantation or primary graft dysfunction was associated with the later development of BOS [41-43]. Daud et al [43] reported that out of 334 lung allograft recipients, 269 had primary graft dysfunction: 130 had grade 1, 69 had grade 2, and 70 had grade 3. A multivariable model demonstrated that the increased risk for BOS with primary graft dysfunction was independent of acute rejection, lymphocytic bronchitis, and community-acquired respiratory viral infections [43]. Furthermore, this increased risk of BOS was directly related to the severity of primary graft dysfunction [43].

### Gastroesophageal reflux

Gastroesophageal (GE) reflux is very common post-lung transplant and may contribute to chronic allograft rejection. The mechanism by which GE reflux contributes to BOS remains unclear. The presence of bile acids and pepsin in bronchoalveolar lavage (BAL) fluid from lung transplant recipients suggests that aspiration may elicit airway injury [44,45]. Moreover, treatment with proton pump inhibitors reduced acid reflux but did not affect nonacid reflux, including bile or pepsin, suggesting the presence of these elements in the lower airways as factors associated with BOS [45]. Early surgical treatment of GE reflux with fundoplication after lung transplantation has been associated with greater freedom from BOS and has improved survival [46,47]. A single institution study reported that 93/128 (73%) of lung transplant recipients had abnormal ambulatory 24-hour esophageal pH probe results [46]. After fundoplication, 16 patients had improved BOS scores, with 13 of these patients no longer meeting the criteria for BOS [46]. Another small study demonstrated that early aggressive surgical treatment of GE reflux with fundoplication not only improved rates of BOS but also survival [47].

### Human leukocyte antigen mismatches

The effect of human leukocyte antigen (HLA) mismatches upon the development of BOS has been reported but remains controversial. The development of anti-HLA class I and II antibodies was associated with BOS [15,48,49]. Furthermore, an association between BOS and mismatches at the A locus [21,50], two DR mismatches [51], or total mismatches at the A locus, B locus, or DR locus [9,50] are reported. However, mismatches at the HLA A locus but not the B locus were associated with acute cellular rejection but not BOS [52]. Further research is needed to investigate this very important issue.

### Autoimmunity

An emerging concept regarding BOS is the possibility of autoimmunity rather than alloimmunity to hidden epitopes of collagen type V. These epitopes are exposed as a result of ischemia and reperfusion injury or other insults that may damage the respiratory epithelium [53]. Further research is ongoing to investigate these important findings.

## Therapies for BOS

### Immunosuppressant therapy

A small number of studies have assessed the different therapeutic modalities that are reportedly beneficial in these patients. Adjustments in immunosuppressant therapy and the use of immunomodulating medications are potential therapeutic options. Adjustments in the immunosuppressive agents have demonstrated some positive outcomes [54-58]. Cairn et al [54] reported that the conversion of cyclosporine to tacrolimus stabilized spirometric measurements in patients with BOS while Whyte et al [55] demonstrated similar results with the introduction of mycophenolate mofetil. In one study, BOS was less likely to progress when sirolimus was substituted for azathioprine in 37 lung transplant recipients receiving cyclosporine or tacrolimus, but the sirolimus had to be discontinued due to side effects [56].

### Novel or emerging therapies

The use of other immunosuppressant therapies in novel ways may improve outcomes for BOS. There is evolving research in the use of aerosolized cyclosporine [59-61]. A single-center, randomized, double-blind, placebo-controlled trial of aerosolized cyclosporine was performed with initiation of the drug within six weeks after lung transplant along with routine systemic immunosuppression [59]. Aerosolized cyclosporine did not improve the rate of acute rejection but improved survival and extended periods of chronic rejection-free survival [59]. More recently, a single center randomized study demonstrated improvement in the pulmonary function of lung transplant patients who received aerosolized cyclosporine for the first 2 years after transplantation compared to placebo [60]. A recent case report demonstrated that aerosolized tacrolimus was associated with improvement in both functional capacity and oxygenation in a patient with BOS [62]. There are other therapies under investigation, including alemtuzumab, an anti-CD 52 antibody, which significantly improved the histological grade of BOS in 7 of 10 patients but had no impact on pulmonary function in an open label study [63].

### Azithromycin therapy

Azithromycin displays immunomodulatory effects that seem to be beneficial in several pulmonary disorders, including BOS. Three studies showed the value of prolonged azithromycin (250 mg orally every other day) in a total of 34 patients with BOS with an improvement in the FEV_1 _for some patients but not all [64-66]. In a larger observational study, Gottlieb et al [67]. demonstrated that 24/81 (30%) patients with BOS had improvement in the FEV_1 _after 6 months of azithromycin therapy; 22 of the 24 responders improved after only 3 months of therapy. With univariate analysis, azithromycin responders at 6 months demonstrated higher pretreatment BAL neutrophils [67]. Neurohr et al [68] also demonstrated that BAL neutrophilia in stable lung transplant recipients had a predictive value in the identification of BOS.

### Statin therapy

Statins (3-Hydroxy-3-methylglutaryl coenzyme A reductase inhibitors) are widely used lipid lowering agents that have demonstrated immunomodulatory effects. The 6-year survival of lung transplant recipients receiving statin therapy was much greater than patients not on statin therapy [69]. Acute rejection was less frequently found in the statin group; none of the 15 recipients started on statin therapy during the first postoperative year developed OB, whereas the cumulative incidence among control subjects was 37%.

### Extracorporeal photopheresis

There is evidence that extracorporeal photopheresis is an effective method of treatment of any inflammatory disorder that is T-cell dependent, including BOS. In the late 1990's, two studies demonstrated the stabilization of airway obstruction due to BOS with extracorporeal photopheresis in 4/5 patients [70] and 5/8 patients [71], respectively, without complications occurring from the procedure. In fact, Salerno et al [71] reported 2 patients having histologic reversal of rejection. Functional stabilization was observed in 3/5 patients with BOS that was accompanied by a slight increase or stabilization of the number of peripheral blood CD4(+)CD25(high) cells with *in vitro *features of Treg cells while the other 2 non-responsive patients with BOS showed a decline in the peripheral Treg subset [72]. An animal study further confirmed that CD4(+)CD25(+) T cells appears to play a key role in the immunomodulatory effects of extracorporeal photopheresis [73]. Over a 10-year period, one study reported that 12 patients with BOS treated with extracorporeal photopheresis had significant improvement in the decline in FEV_1_, 112 mL/month before therapy and 12 mL/month after 12 cycles of therapy (P = 0.011) [74]. The effect of extracorporeal photopheresis on absolute FEV_1 _on the group of 12 patients was not significant and the therapy was tolerated [74].

More recently, 60 lung transplant recipients experienced a reduction in the rate of decline in lung function associated with progressive BOS with extracorporeal photopheresis therapy [75]. The decline in FEV_1 _6 months prior to treatment with extracorporeal photopheresis was 116.0 mL/month, but the slope decreased to 28.9 mL/month during the 6-month period after initiation of therapy with the mean difference in the rate of decline being 87.1 mL/month (P < 0.0001) [75]. Furthermore, the FEV_1 _actually improved in 25.0% of patients after starting extracorporeal photopheresis with a mean increase of 20.1 mL/month [75].

## Management Strategies in BOS

An important therapeutic strategy in treating BOS is initial prevention and aggressive treatment of known associated factors, as well as early identification of BOS in order to immediately begin available therapies. Initially, the clinical management of these patients should focus on risk reduction of primary graft dysfunction by decreasing mechanical ventilation time for donors and reducing allograft ischemia time, while also limiting cardiopulmonary bypass and blood product transfusions during transplantation [76].

**Figure 2:**
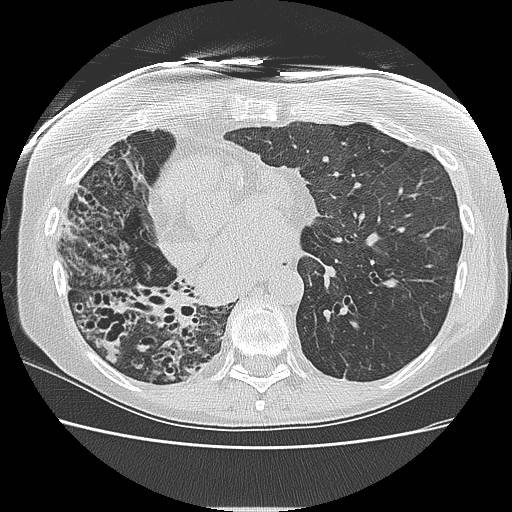
**High resolution CT scan of the chest demonstrating bilateral bronchiectasis (right more severe than left) in a patient who underwent right single lung transplantation in 1992 for alpha-1-antitrypsin deficiency and left single lung transplantation in 2003 for bronchiolitis obliterans syndrome**.

Routine screening to define the onset of BOS is very important as there appears to be a therapeutic window for some of the treatment options available. Jain et al [77] demonstrated that azithromycin treatment initiated before the development of BOS stage 2 was independently associated with a significant reduction in the risk of death. Thus, clinicians should be closely monitoring lung transplant recipients, carefully monitoring for early chronic rejection. Spirometry should be performed routinely on lung transplant recipients, looking for any changes in the FEV_1 _and FEF_25-75% _measurements based on the ISHLT classification system (Table 1). The use of HRCT imaging with inspiratory and expiratory views of the chest to assess for airtrapping may be helpful based on initial studies [18,78], but further research is less conclusive regarding its value [79-81]. Currently, radiographic imaging remains supportive in the diagnostic evaluation and management of BOS. Figure 2 demonstrates the usefulness of HRCT imaging in diagnosing BOS in a 55 year-old patient who underwent right single lung transplantation in 1992 for alpha-1-antitrypsin deficiency but suddenly developed a 25% reduction in FEV_1 _3 years after undergoing single left lung transplantation for BOS. The right allograft clearly had significant bronchiectasis due to long-standing BOS, but the more recent allograft on the left side had signs of bronchiectasis with airtrapping, further supporting the diagnosis of BOS in that allograft.

Aggressive treatment of GE reflux, avoidance of infection, and timely vaccinations are instrumental in managing lung transplant recipients. Experimental risk factors reported in BOS should be considered from a clinical standpoint during the evaluation of transplant recipients, including higher bronchoalveolar (BAL) neutrophilia and IL-8 levels [82,83] as well as airway colonization with *Pseudomonas aeruginosa *[84,85]. Further research is needed to better define the clinical role of these evolving factors.

## Retransplantation for BOS

The definitive treatment for BOS and resulting bronchiectasis is retransplantation. However, lung retransplantation remains very controversial due to limited organ availability and lower survival rates as compared to initial transplants. In 1995, Novick et al [86] reviewed the records of 72 patients who underwent retransplantation for BOS at 26 North American and European centers. In this cohort, the actuarial survival rates were 71% at 1 month, 43% at 1 year, and 35% at 2 years [86]. For the 90-day postoperative survivors, 63% were alive 2 years after retransplantation [86]. Further study in larger cohorts of 139 retransplant recipients in 1995 and 230 retransplant recipients in 1998 demonstrated very similar survival statistics [87,88]. Although survival rates for lung retransplantation were lower than survival rates for initial transplants, lung retransplantation continued to be performed in recipients who developed BOS. More recently, survival rates after lung retransplantation have improved [89-94]. A retrospective cohort study of 205 patients who underwent lung retransplantation between January 2001 and May 2006 in the United States demonstrated a 1-year survival of 62%, 3-year survival of 49%, and 5-year survival of 45% [89]. These authors did not assess the outcomes of patients undergoing retransplantation specifically for BOS, but there was definite improvement in outcomes for all patients after lung retransplantation in the modern era. Moreover, there have been smaller studies that have addressed the survival of lung retransplantation for BOS in adult patients; Table 3 outlines these research studies published since 2000. These 5 recent studies report 1-year and 5-year survival rates at 60-75% and 44-62%, respectively in comparison to the current unadjusted survival rates for initial transplants of 79% at 1 year and 52% at 5 years as published by Christie et al [4].

**Table 3:** Lung retransplantation for bronchiolitis obliterans syndrome.

First Author	Mean Age (years)	Number of Patients	Survival	Citation
Brugière et al	50	15	60% (1-year) 53% (2-year) 45% (5-year)	73

Martinu et al	40	12	75% (1-year)	74

Strueber et al	42	37	78% (1-year) 62% (5-year)	75

Aigner et al	36	19	72% (1-year) 61% (5-year)	76

Osaki et al	44	12*	67% (1-year) 67% (2-year) 44% (5-year)	77

*1 patient underwent retransplantation twice

## Conclusions

For lung transplant recipients, BOS remains to be the primary cause of mortality after the first year. In the current lung allocation score era of lung transplantation, recipients have significantly fewer BOS-free days after 3-year follow-up [95]. Further research is needed to better define the pathophysiologic mechanisms in BOS in order to either prevent or delay onset of the disorder. The therapies available for BOS currently are very limited and serve only to slow the decline in pulmonary function. Lung retransplantation continues to be controversial, but survival rates have improved in patients with BOS over the past decade and thus should be considered as a treatment option in this patient population.

## List of Abbreviations

A list of abbreviations used in this manuscript in alphabetical order are: (BOS): bronchiolitis obliterans syndrome; (BAL): bronchoalveolar; (COPD): chronic obstructive pulmonary disease; (CMV): Cytomegalovirus; (EBV): Epstein-Barr virus; (FEF_25-75%_): forced expiratory flow during the middle half of the forced vital capacity; (FEV_1_): forced expiratory volume in 1 second; (GE): gastroesophageal; (HRCT): high resolution computed tomography; (ISHLT): International Society for Heart and Lung Transplant; (OB): obliterative bronchiolitis; and (TBB): transbronchial biopsies.

## Competing interests

The author declares that they have no competing interests.

## Authors' contributions

The author of this manuscript completed the literature review and developed the manuscript without assistance. There were no contributors in the preparation and development of this manuscript. No funding was required to complete this work.
